# Assessment of metabolic syndrome in infertile women with polycystic ovary syndrome in a rural population of South India: A cross-sectional study

**DOI:** 10.18502/ijrm.v20i3.10707

**Published:** 2022-04-21

**Authors:** Sreelalitha Kayali, Thyagaraju Chitra, Sadishkumar Kamalanathan, Hanumanthappa Nandeesha

**Affiliations:** ^1^Department of Obstetrics and Gynecology, Jawaharlal Institute of Postgraduate Medical Education and Research, Puducherry, India.; ^2^Department of Endocrinology, Jawaharlal Institute of Postgraduate Medical Education and Research, Puducherry, India.; ^3^Department of Biochemistry, Jawaharlal Institute of Postgraduate Medical Education and Research, Puducherry, India.

**Keywords:** Polycystic ovary syndrome, Metabolic syndrome, Hyperandrogenism, Hormones.

## Abstract

**Background:**

Polycystic ovary syndrome (PCOS) is known to be associated with metabolic syndrome (MS). It is characterised by insulin resistance, hyperinsulinemia, dyslipidemia, obesity and hypertension. Data related to MS in infertile women with PCOS are limited in Indian populations.

**Objective:**

This study aims to compare the prevalence of MS in infertile women with and without PCOS in a rural population in South India.

**Materials and Methods:**

130 women with PCOS and 130 women without PCOS were enrolled in this cross-sectional study. A detailed history was taken and a physical examination was done for all women. Anthropometric parameters, a glucose tolerance test, fasting glucose / insulin levels, trigylcerides, high-density lipoprotein cholesterol and blood pressure were assessed in all participants. The International Diabetes Federation criteria were applied for assessment of MS.

**Results:**

MS was more prevalent in infertile women with PCOS (42.3%) compared to women without PCOS (19.3%). 56.9% of women with PCOS had low high-density lipoprotein cholesterol levels, 46.2% had high triglycerides, 71.5% had a high waist circumference, 31.5% had high blood pressure and 37.7% had high blood glucose levels. 26.0% of the women with PCOS had a healthy weight, and MS was seen in 6.9% of these women.

**Conclusion:**

The prevalence of MS was higher in women with PCOS in comparison to women without PCOS. Among the women with PCOS, the prevalence of MS increased with age ( > 27 yr), body mass index and waist circumference (71.5%), and even healthy women with PCOS contributed to 7% of MS. Hence it becomes necessary to screen all women with PCOS for metabolic profile risk factors at young age itself to prevent long term cardiovascular complications.

## 1. Introduction

Polycystic ovary syndrome (PCOS) is a highly prevalent disorder in India, known to affect about 20-30% of adolescent women (1-3). The presence of PCOS is linked with an increased prevalence of type 2 diabetes mellitus (DM), cardiovascular disease, and metabolic syndrome (MS) (4-6). MS is characterized by insulin resistance, hyperinsulinemia, dyslipidemia, obesity and hypertension (7, 8). Insulin resistance is considered to play a key role in the development of PCOS and related conditions (9). Previous studies have identified that insulin resistance and central adiposity affect not only obese but also healthy-weight women with PCOS, leading them to develop MS (4, 9, 10). In the last two decades, many researchers have attempted to better understand the mechanisms behind the development of PCOS and its impact on females, particularly in relation to infertility and insulin resistance (5, 6).

Several studies have documented that the prevalence of MS among PCOS women is between 14.5% and 47.3% (11-16). Genetic factors, diet and lifestyle habits are known to influence the prevalence of MS in women with PCOS (17, 18).

Since the data about the prevalence of MS in women with PCOS in Indian rural populations are limited, the present study was designed to compare MS in infertile women with and without PCOS.

## 2. Materials and Methods

This cross-sectional study was conducted in a tertiary care hospital, Pondicherry, India from March 2016 to February 2018. 130 infertile women with PCOS and 130 without PCOS in the age range of 21-35 yr were included in the study. PCOS was diagnosed according to the Rotterdam 2003 criteria (19).

### Sample size calculation

According to previous studies, the prevalence of MS in women with and without PCOS is 37.5% and 19.5%, respectively (20-22). The expected difference in the prevalence of MS between infertile women with and without PCOS was 18.0% (37.5% vs. 19.5%). The sample size was estimated to be 260 (130 in each group) by using the standard statistical formula comparing two independent proportions at a significance level of 5% and a power of 90%.

### Methodology

Women attending an infertility clinic, according to inclusion criteria, were considered as participants.

The basic inclusion criteria were:

Infertile women with PCOS (aged 21-35 yr) according to the Rotterdam 2003 criteria, i.e., any two of the below:

Clinical hyperandrogenism (Ferriman-Gallwey score > 8) or biochemical hyperandrogenism;

Oligomenorrhea (fewer than 6-9 menses per yr) or oligo-ovulation; and/or

Polycystic ovaries on ultrasound ( ≥ 12 antral follicles in one ovary or ovarian volume ≥ 10 cm^3^).

Women with thyroid disorders, congenital adrenal hyperplasia, adrenal tumours or Cushing's syndrome, and those who were on steroid therapy or oral contraceptive pills for more than three months were excluded from the study.

A standard questionnaire was used to document a detailed history and medical information. Menstrual history (regular/irregular), and medical and family history of diabetes, obesity, ischemic heart disease, and hypertension were recorded. 5 ml of fasting blood samples were taken from the participants for evaluation of lipid profile, insulin levels, testosterone levels and blood sugar levels. Two hr postprandial samples were taken for blood glucose and insulin levels. For each participant, BMI was calculated from measured weight (kg) and height (m) using the formula BMI = kg/m^2^. The waist hip ratio was calculated by dividing waist measurement (W) by hip measurement (H). Blood pressure was measured in millimeters of mercury (mmHg) by using standard mercury sphygmomanometer in right arm, on two occasions with 15 min gap between measurements.

Signs of androgen excess like hirsutism, acne, alopecia, and insulin resistance were recorded. For considering hirsutism, a Ferriman-Gallwey score > 8 was considered (23). Alopecia is thinning of the hair, which can be due to hyperandrogenism in PCOS. Dihydrotestosterone, a metabolite of androgen, binds to receptors in the scalp follicles and shrinks those follicles making it impossible for healthy hair to survive. Oligomenorrhea was considered as the presence of three or more cycles of greater than 35 days in the previous six months, and amenorrhea was defined as the absence of vaginal bleeding for three months. Transvaginal ultrasound was done for all women on second day of menstrual cycle for both ovaries. Furthermore, a 75 gr fasting and 2-hr oral glucose tolerance test were done for estimating blood glucose and insulin levels. Subsequently, fasting blood samples were used for estimation of levels of trigylcerides, low density lipoproteins, high density lipoprotein (HDL), and cholesterol.

The criterion proposed by International Diabetes Federation (IDF), (which is based on waist circumference, triglycerides, high density lipoprotein-cholesterol, fasting glucose and blood pressure) was considered in this study.

### Criteria of MS

The criteria proposed by the IDF diagnose MS (24, 25) as women with a waist circumference ≥ 80 cm plus any two of the following four parameters:

Raised triglycerides ( ≥ 150 mg/dl or a history of specific treatment for this lipid abnormality);

Raised BP (Systolic BP ≥ 130 mmHg or diastolic BP ≥ 85 mmHg, or on treatment for previously diagnosed hypertension);

Reduced HDL cholesterol ( < 50 mg/dl or a history of specific treatment for this lipid abnormality); and/or

Raised fasting blood glucose ( ≥ 100 mg/dl or a previous diagnosis of type 2 DM).

### Ethical considerations

The present study was approved by the JIPMER Ethics Committee for Human Studies, Puducherry, India (Code: JIP/IEC/SC/2016/25/852). Written informed consent was obtained from all participants before starting the study.

### Statistical analysis 

The analysis was done using the Statistical Package for the Social Sciences (SPSS) software version 22 (IBM SPSS, USA). Descriptive statistics were expressed as mean and standard deviation or median (range) as appropriate. Frequency and percentage were used to describe the characteristics of the participants. Outcome measures like MS were expressed as proportions. Chi-square test was used to compare proportions across the two groups. Biochemical parameters such as age, weight, and BMI were compared using the Mann-Whitney U test (for non-normally distributed data) between the two groups.

## 3. Results

The comparison of the clinical, hormonal, and metabolic characteristics of the infertile women with and without PCOS is shown in table I. Primary infertility constituted around 90% in the PCOS group compared to 82% in the non-PCOS group. Women in the PCOS group had more irregular cycles (74% vs. 17%), hyper-androgenism signs (33% vs. 13%) and insulin resistance (21% vs. 0%) in comparison to the non-PCOS group which was statistically significant (Table I).

96 women (74.0%) in the PCOS group were overweight or obese compared to 17.6% in the non-PCOS group. Fasting insulin, homeostatic model assessment of insulin resistance, BMI, waist circumference, BP, and triglycerides were significantly higher and HDL cholesterol was significantly lower in women with PCOS compared to without PCOS.

The prevalence of MS in women with PCOS is shown in table II. 42.3% of women with PCOS (n = 55) were found to have MS, whereas, among women without PCOS, only 19.2% (n = 25) were found to have MS. As shown in table III, in the PCOS group, 71.5% had a waist circumference of ≥ 80 cm, 31.5% had systolic BP ≥ 130 mmHg or diastolic BP ≥ 85 mmHg, 37.7% had fasting blood glucose ≥ 5.6 mmol/L, 46.2% had triglycerides ≥ 1.7 mmol/L, and 56.9% had HDL cholesterol ≤ 1.3 mmol/L. 11.0% of women with PCOS had a BMI < 25 kg/m^2^, and of these, nine women (6.9%) had MS, according to the IDF criteria.

The clinical, metabolic, and hormonal characteristics of the women with PCOS with and without MS are analyzed in table III. Age, BMI, waist circumference, free testosterone, 2-hr insulin levels, acne, acanthosis, and hirsutism were significantly higher in women with PCOS who had MS compared to those without MS.

The prevalence of MS was 56.25% in women with a BMI > 30 kg/m^2^ and 11.34% in the women with a BMI < 25 kg/m^2^ (Table IV).

**Table 1:** Comparison of clinical, hormonal, and metabolic characteristics of women with and without PCOS


**Variables**		**PCOS group (n = 130)**	**Non-PCOS group (n = 130)**	**P-value**
**Clinical**				
	**Age (yr)**	27.29 ± 4.26	27.22 ± 2.83	< 0.01*
	**BMI (kg/m^2^)**	26.80 ± 2.62	22.90 ± 2.62	< 0.01*
	**Waist circumference (cm)**	85.00 ± 11.13	78.73 ± 7.59	< 0.01*
	**Infertility (primary/secondary)**	118/12	107/23	
	**Duration of infertility (yr)**	3.44 ± 0.88	3.62 ± 0.94	
	**Irregular menstrual cycles (%)**	97 (74.62%)	22 (16.92%)	
	**Hirsutism (%)**	44 (33.85%)	17 (13.08%)	< 0.01**
	**Acne (%)**	51 (39.23%)	21 (16.15%)	< 0.01**
	**Alopecia (%)**	3 (2.31%)	0	< 0.01**
	**Acanthosis nigricans (%)**	28 (21.54%)	0	< 0.01*
	**Systolic blood pressure (mmHg)**	115.15 ± 11.63	107.31 ± 9.55	< 0.01*
	**Diastolic blood pressure (mmHg)**	73.32 ± 7.50	70.54 ± 5.18	< 0.01*
**Hormonal**				
	**Free testosterone (μg/L)**	1.10 ± 0.43	0.43 ± 0.07	< 0.01*
	**Fasting plasma glucose (mmol/L)**	5.34 ± 0.89	5.00 ± 0.88	< 0.01*
	**Post prandial glucose (mmol/L)**	6.57 ± 1.26	5.84 ± 1.14	< 0.01*
	**HOMA IR**	2.35 ± 0.21	1.41 ± 0.39	< 0.01*
	**Fasting plasma insulin (mIU/L)**	14.36 ± 7.22	8.00 ± 4.71	< 0.01*
	**Two hr plasma insulin (mIU/L)**	39.95 ± 9.73	19.67 ± 6.04	< 0.01*
	**Triacylglycerol (mmol/L)**	1.74 ± 0.33	1.41 ± 0.30	< 0.01*
	**HDL cholesterol (mmol/L)**	1.18 ± 0.25	1.43 ± 0.22	< 0.01*
	**Sex hormone binding globulin (nmol/L)**	51.89 ± 8.92	87.96 ± 13.02	< 0.01*

*Data presented as Mean ± standard deviation and analyzed by Mann-Whitney U test. **Data presented as frequency (percentage) and analysed by Chi-square test. PCOS: Polycystic ovary syndrome, HOMA IR: Homeostatic model assessment of insulin resistance, HDL: High-density lipoprotein, BMI: Body mass index

**Table 2:** Prevalence of MS in the women with and without PCOS based on IDF criteria


**Variables**	**Women with PCOS**	**Women without PCOS**
**Waist circumference ≥ 80**	93 (71.5%)	45 (34.6%)
**Trigylcerides ≥ 1.7 mmol/L or treatment**	60 (46.2%)	35 (26.9%)
**HDL < 1.3 mmol/L or treatment**	74 (56.9%)	30 (23.1%)
**Blood pressure ≥ 130/85 mmHg**	24 (18.5%)	6 (4.6%)
**Fasting glucose ≥ 5.6 mmol/L or previously diagnosed type 2 DM**	49 (37.7%)	36 (27.7%)
**MS**	55 (42.3%)	25 (19.3%)

PCOS: Polycystic ovary syndrome, HDL: High-density lipoprotein, DM: Diabetes mellitus, MS: Metabolic syndrome, IDF: International Diabetes Federation

**Table 3:** Clinical and biochemical parameters of women with PCOS with and without MS


**Variables**	**PCOS group with MS (n = 55)**	**PCOS group without MS (n = 75)**	**P-value**
**Age (yr)**	29.20 ± 4.83	25.88 ± 3.15	< 0.01*
**BMI (kg/m^2^)**	27.58 ± 2.44	26.22 ± 2.61	< 0.01*
**Waist circumference (cm)**	89.07 ± 6.19	82.01 ± 12.91	< 0.01*
**Free testosterone (μg/L)**	1.32 ± 0.55	0.94 ± 0.23	< 0.01*
**Fasting insulin (mIU/L)**	14.56 ± 5.98	14.22 ± 8.05	0.79*
**Two hr insulin (mIU/L)**	43.62 ± 9.40	37.26 ± 9.13	< 0.01*
**HOMA IR**	2.43 ± 0.20	2.29 ± 0.20	< 0.01*
**Acne**	27 (49.1%)	24 (32.0%)	< 0.05**
**Acanthosis nigricans**	25 (45.5%)	19 (25.3%)	0.02**
**Hirsutism**	18 (32.7%)	10 (13.3%)	0.01**

*Data presented as Mean ± standard deviation and analyzed by Mann-Whitney U test. **Data presented as frequency (percentage) and analysed by Chi-square test. PCOS: Polycystic ovary syndrome, MS: Metabolic syndrome, HOMA IR: Homeostatic model assessment of insulin resistance, BMI: Body mass index

## 4. Discussion

In our study, we found that the prevalence of MS was higher in women with PCOS compared to women without PCOS. Advanced age is considered as important risk factor for development of MS (11). In our study, mean age of women who developed MS was 29.20 ± 4.83, which is in agreement with other studies (15, 20). We also observed that hirsutism, acne, alopecia, insulin, and testosterone were higher in women with PCOS who had MS.

PCOS is characterized by alteration in endocrine and metabolic parameters. The common metabolic abnormalities in PCOS and MS are hyperandrogenism and insulin resistance (26). In our study, hormones like testosterone, sex hormone binding globulin, fasting insulin, and homeostatic model assessment of insulin resistance were significantly higher in the PCOS group compared with non-PCOS group. These findings are in agreement with earlier studies from Park et al., Mandrelle et al., Luotola et al. (11, 20, 27). Insulin resistance along with elevated circulating insulin levels induces increased androgen production from the theca cells and abnormal changes in the lipid metabolism. Further, androgen excess supports the presence of unfavourable metabolic state leading to a central distribution of fat and dyslipidemia. Metabolic derangements were seen more in obese PCOS women in our study which was similar to a study done by Li and co-workers (28), who found a higher level of free androgen index, higher prevalence of insulin resistance, and lower sex hormone binding globulin (SHBG) levels in their PCOS cohort group. When abdominal adipose tissue is broken, there is an observation of rise in free fatty acid levels in portal circulation, which leads to chronic hyperinsulinemia. Free fatty acids may impair the hepatic extraction of insulin. As mentioned in previous studies (27, 28, 29), insulin resistance is one of the features in the development of PCOS, which provides additional support to explain why obesity worsens the symptoms of PCOS.

In our study, the overall prevalence of MS in PCOS women was 42.3% in comparison to 19.3% in women without PCOS. These findings were similar to the previous studies on Indian women with PCOS (both adolescents as well as adults) (25, 29), with prevalence ranging from 41-46% but in contrast to a study done by Park and colleagues (11) and Mafaldo Soares and co-workers (15), who showed prevalence of MS ranging from 25-28%. The difference in low prevalence of MS might be due to different ethnicity, substantial proportion of participants were normal weight and NCEP criteria were used for diagnosis of MS in this cohort study (11).

In our study, mean and standard deviation of waist circumference (89.07 ± 6.19) in PCOS women who developed MS were higher compared with women without MS (82.01 ± 12.91). These findings were similar with other studies (11, 15, 30), which have shown that increased waist to hip ratio is a better predictor of development of MS in PCOS women compared to other metabolic parameters including BMI, which is in agreement with our study which showed that the MS prevalence rate increased with age and obesity. The prevalence of MS in our study also increased with an increase in BMI, which is in accordance with previous studies (Table IV).

Even though obesity is considered to increase the risk of PCOS and MS, in the current study, 26.2% (n = 34) of women with PCOS were found to be of a healthy weight and within this group, nine women (6.9%) had MS (10.8% had a waist circumference of more than 80 cm and 12.3% had triglyceride levels of more than or equal to 1.7 mmol/L). This indicates the need for evaluation of MS even in healthy-weight women with PCOS. Previous studies have attributed the high prevalence of MS in women with PCOS to the pattern of fat distribution which leads to hyperandrogenism, insulin resistance, and dyslipidemia (31, 32). Comparison of the prevalence of MS in subjects with PCOS with various other studies is depicted in table V.

**Table 4:** Comparison of the distribution of MS according to BMI in this study vs. other studies


**Authors (Ref)**	**Sample size**	**Study type**	**BMI < 25 **	**25 ≤ BMI ≤ 29.9 **	**BMI > 30 **
**Mafaldo Soares ** ***et al.*** ** (15)**	102 Brazilian women with PCOS	Cross-sectional	3.2%	19.2%	52.2%
**Mandrelle ** ***et al.*** ** (20)**	120 women with PCOS in South India	Cross-sectional	15.7%	51.9%	60.0%
**Abdelazim ** ***et al.*** ** (33)**	220 infertile women with PCOS in Kuwait	Cross-sectional	22.6%	34.1%	46.9%
**Apridonidze ** ***et al.*** ** (34)**	106 PCOS women	Retrospective	18.28%	31.33%	50.1%
**Current study**	130 women with PCOS and 130 women without PCOS in South India	Cross-sectional	11.34%	52.87%	56.25%

MS: Metabolic syndrome, BMI: Body mass index (kg/m^2^), PCOS: Polycystic ovary syndrome

**Table 5:** Comparison of the prevalence of MS in women with PCOS with various other studies


**Authors (Ref)**	**Study type**	**Studied population**	**MS prevalence in PCOS vs. controls**
**Glueck ** ***et al.*** ** (12)**	Prospective	138 PCOS NIH, mBMI: Obese No controls Caucasians	NCEP: 46.4%
**Apridonidze ** ***et al.*** ** (34)**	Retrospective	106 PCOS NIH, mBMI: Obese reference group: Age-matched, lower BMI multiracial	NCEP: 43%
**Mafaldo Soares ** ***et al.*** ** (15)**	Cross-sectional	102 PCOS (R), mBMI: Overweight No controls Brazilian	NCEP: 28.4%
**Park ** ***et al.*** ** (11)**	Cross-sectional	113 PCOS (NIH), mBMI: Lean Korean	NCEP: 14.5% (3.5-fold higher than in age-matched women in Korean urban population)
**Cheung ** ***et al.*** ** (17)**	Cross-sectional	295 PCOS (R), mBMI: Overweight 98 older controls, lower BMI Chinese	NCEP: 24.9% vs. 3.1% BMI-matched overweight/obese: 41.3% vs. 15% BMI-matched lean: No difference
**Bhattacharya ** ***et al.*** ** (25)**	Cross-sectional	117 PCOS (R) (78 adults and 39 adolescents), mBMI: Overweight Indian	IDF: 46.2%
**Our study**	Cross-sectional	130 PCOS (Rotterdom's Criteria) 130 controls	IDG criteria: Prevalence of MS in PCOS group is 42.3% compared to 19.3% in non-PCOS group

The above studies were conducted in adult women unless else stated. In those studies that included mixed BMI groups of patients, the characterization of PCOS subjects as lean (BMI < 25 kg/m^2^), overweight (BMI: 25-30 kg/m^2^) or obese (BMI > 30 kg/m^2^) was based on the mean BMI value (mBMI) of each studied population. MS: Metabolic syndrome, PCOS: Polycystic ovary syndrome, BMI: Body mass index, IDF: International Diabetes Federation, IDG: International Diabetes Guidelines, NCEP: National cholesterol education program adult treatment panel III criteria

### Limitations

One of the limitations of the study was that the women with PCOS were not followed up to assess the impact of MS on maternal and fetal outcomes. The cause-effect relationship between PCOS and MS was not established owing to the cross-sectional design of the study.

## 5. Conclusion

The prevalence of MS was higher in women with PCOS in comparison to the women without PCOS. Among the women with PCOS, the prevalence of MS increased with age, BMI, and waist circumference more than 80 cm, and even PCOS with normal weight contributed to 7% of MS. These findings suggest that the formulation of a screening policy for all women with PCOS for any metabolic risk factors is needed, and to educate them regarding long term complications, and the need for appropriate diet and lifestyle modifications.

##  Conflict of Interest

The authors declare that there is no conflict of interest.
